# Few-layer HfS_2_ transistors

**DOI:** 10.1038/srep22277

**Published:** 2016-03-01

**Authors:** Toru Kanazawa, Tomohiro Amemiya, Atsushi Ishikawa, Vikrant Upadhyaya, Kenji Tsuruta, Takuo Tanaka, Yasuyuki Miyamoto

**Affiliations:** 1 Department of Physical Electronics, Tokyo Institute of Technology, 152-8552 Japan; 2 Quantum Nano Electronics Research Center, Tokyo Institute of Technology, 152-8552 Japan; 3 Metamaterials Laboratory, RIKEN, 351-0198, Japan; 4 Department of Electrical & Electronic Engineering, Okayama University, 700-8530, Japan; 5 Innovative Photon Manipulation Research Team, RIKEN, 351-0198, Japan; 6 Interdisciplinary Graduate School of Science and Engineering, Tokyo Institute of Technology, 226-8502, Japan

## Abstract

HfS_2_ is the novel transition metal dichalcogenide, which has not been experimentally investigated as the material for electron devices. As per the theoretical calculations, HfS_2_ has the potential for well-balanced mobility (1,800 cm^2^/V·s) and bandgap (1.2 eV) and hence it can be a good candidate for realizing low-power devices. In this paper, the fundamental properties of few-layer HfS_2_ flakes were experimentally evaluated. Micromechanical exfoliation using scotch tape extracted atomically thin HfS_2_ flakes with varying colour contrasts associated with the number of layers and resonant Raman peaks. We demonstrated the I-V characteristics of the back-gated few-layer (3.8 nm) HfS_2_ transistor with the robust current saturation. The on/off ratio was more than 10^4^ and the maximum drain current of 0.2 μA/μm was observed. Moreover, using the electric double-layer gate structure with LiClO_4_:PEO electrolyte, the drain current of the HfS_2_ transistor significantly increased to 0.75 mA/μm and the mobility was estimated to be 45 cm^2^/V·s at least. This improved current seemed to indicate superior intrinsic properties of HfS_2_. These results provides the basic information for the experimental researches of electron devices based on HfS_2_.

To realise ultra-low power circuits, three important electrical characteristics are desirable in channel materials for field-effect transistors (FETs). They are low supply voltage, high drivability, and small off-leakage current. The first two can be achieved by channel scaling in the same manner as that in previous techniques^1^. On the other hand, continuous scaling of channel length L_ch_ can lead to a severe short-channel effect (SCE)^2^. SCE increases the off-leakage current and standby power consumption of logic circuits. One of the possible solutions to reduce SCE in ultra-scaled channel (L_ch_ < 10 nm) is the introduction of atomically thin body channel structure. However, in an extremely thin body channel consisting of conventional semiconductors such as silicon (Si)^3^, InGaAs^4^, and germanium^5^, surface roughness scattering severely reduces the carrier mobility^6^ and ballistic coefficient. Two-dimensional (2D) materials have layered crystal structure with strong covalent/ionic bond in a plane and a weak Van der Waals interaction between layers. From this unique property, 2D materials can obtain an atomically flat surface with discrete thickness defined by its single-layer thickness, a carrier transport with minimal surface roughness scattering and a strong immunity to short channel effect. These 2D material features can be useful in achieving ultra-low power operation because of the realisation of high mobility with extremely short and thin channel design. Another critical property for low leakage current operation is the bandgap value. Graphene^7^,^8^, which is the most famous 2D material, has no bandgap without the application of additional techniques^9^,^10^, and this property is a major hindrance in reducing the drain leakage. Zero (or very small) bandgap materials such as graphene show ambipolar behaviour in field-effect current modulation. The off current could not be small enough due to reverse polarity operation. Although, this property is useful in many applications (e.g. RF applications^11^ and sensing^12^), it becomes impractical in low-power logic circuits^13^. Moreover, to reduce the drain leakage current due to band-to-band tunnelling such as gate-induced drain leakage^14^, wide bandgap is desirable for materials with finite bandgap. Therefore, 2D materials with a finite bandgap, such as transition metal dichalcogenides (TMDs) (e.g. MoS_2_^15^,^16^ and WSe_2_^17^,^18^) or phosphorene^19^,^20^, are required for low power consumption FETs. The mobility and bandgap should be carefully and extensively investigated for various applications and for performance improvement.

HfS_2_ (Hafnium disulphide) was previously considered as a semi-insulating material because the measured conductivity of HfS_2_ is very low compared with that of MoS_2_^21^,^22^. However, in recent studies, some attractive properties of single-layered HfS_2_ have been theoretically predicted. The long-wave acoustic phonon limited mobility μ_AP_ of TMDs estimated in ref. 23 and the energy bandgap E_g_ in ref. 24 are shown in Fig. 1, which indicate that a substantial trade-off exists between μ_AP_ and E_g_. Single-layer HfS_2_ is expected to have good upper limit of mobility (~1800 cm^2^/V∙s) and reasonable energy bandgap (~1.2 eV) for a high on/off ratio. Although several reports concerning the electron mobility of MoS_2_^16^,^25^,^26^ suggested the existence of several other scattering mechanisms, we understand that comparison of the mobilities in TMDs using the uniform calculation method will aptly represent the relative trend among these mobilities. Additionally, large electron affinity of HfS_2_ has a possibility to achieve low contact resistance for n-type carrier transport because of basic principle of the contact formation between semiconductors and metals. Consequently, we planned to experimentally evaluate the FET performance of HfS_2_ and reveal its potential for transistor channel. In addition, the other properties of thin-layered HfS_2_ have not been thoroughly investigated, and we report some basic characteristics of atomically thin-layered HfS_2_ essential for fabrication and characterisation.

## Results and Discussion

### Estimation of the ballistic current for single-layer HfS_2_

**Figure 2:**
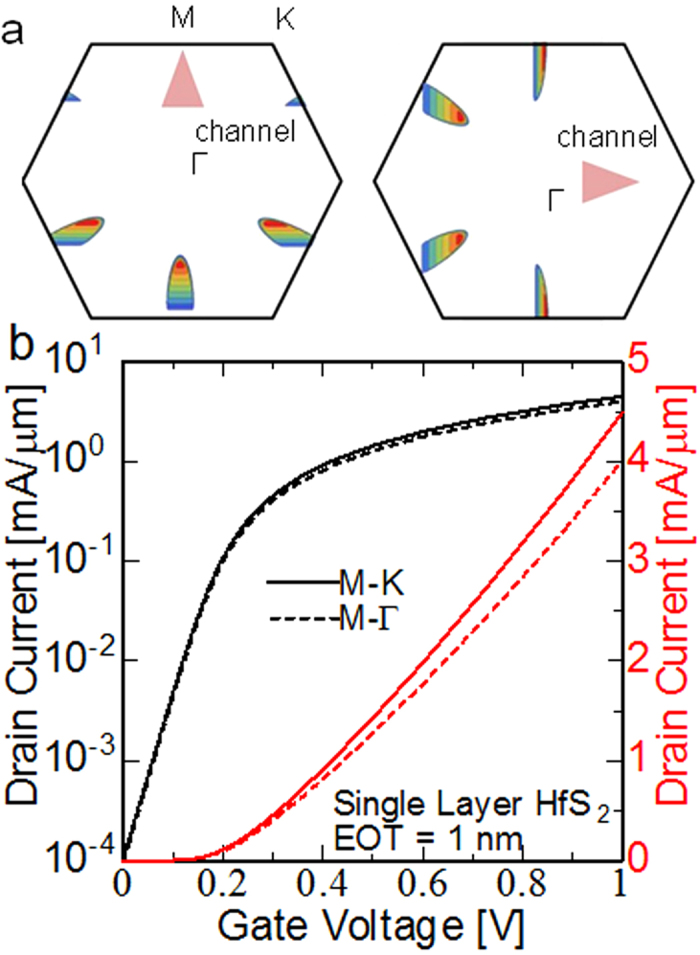
Calculated transport properties of single-layer HfS_2_. (**a**) Current distribution in the 2D Brillouin zone. (**b**) Transfer characteristics at the ballistic limit. Two different directions are considered, but no significant difference in the total current is observed because the threefold M valleys are mixed.

Prior to the experiment, the ballistic current of a single-layer HfS_2_ FET was calculated using a simple numerical method at the potential bottleneck^27^ to evaluate the potential of the HfS_2_ channel. The electron effective masses, which are essential for this numerical calculation, were 0.24 m_0_ along the M–K and 3.3 m_0_ along the M–Γ^24^. The several assumptions used in the ballistic model were as follows: (1) the electron backscattering in the channel and drain injection is negligible for the ballistic limit. Therefore, only half of the states with positive wave numbers are considered. (2) The electron distribution thickness in the HfS_2_ layer is not considered. In other words, all electrons exist at the interface between the oxide and HfS_2_. (3) Parabolic and ellipsoidal conduction bands are assumed for threefold valleys at the M points in the Brillouin zone. (4) The equivalent oxide thickness is defined to be 1 nm. Further details of calculations are described in supplementary information. Figure 2(a) shows the current distribution in the 2D first Brillouin zone by the contour plot at 300 K. The arrows in the inset denote the direction of the channel selected with high symmetry (M–Γ and M–K). Figure 2(b) shows the calculated transfer characteristics of single-layer HfS_2_ at room temperature (RT). The off current at a gate voltage of 0 V was defined to be 100 nA/μm. Although the electrons in the conduction band had strong anisotropy in the effective masses, the drain current did not indicate a significant difference in the channel direction because of the mixing of the threefold valleys with an angle of 120° from one another. It reached to 2 mA/μm at the gate voltage of 0.6 V.

### Basic properties of HfS_2_ flakes micromechanically transferred on Al_2_O_3_/Si

**Figure 3:**
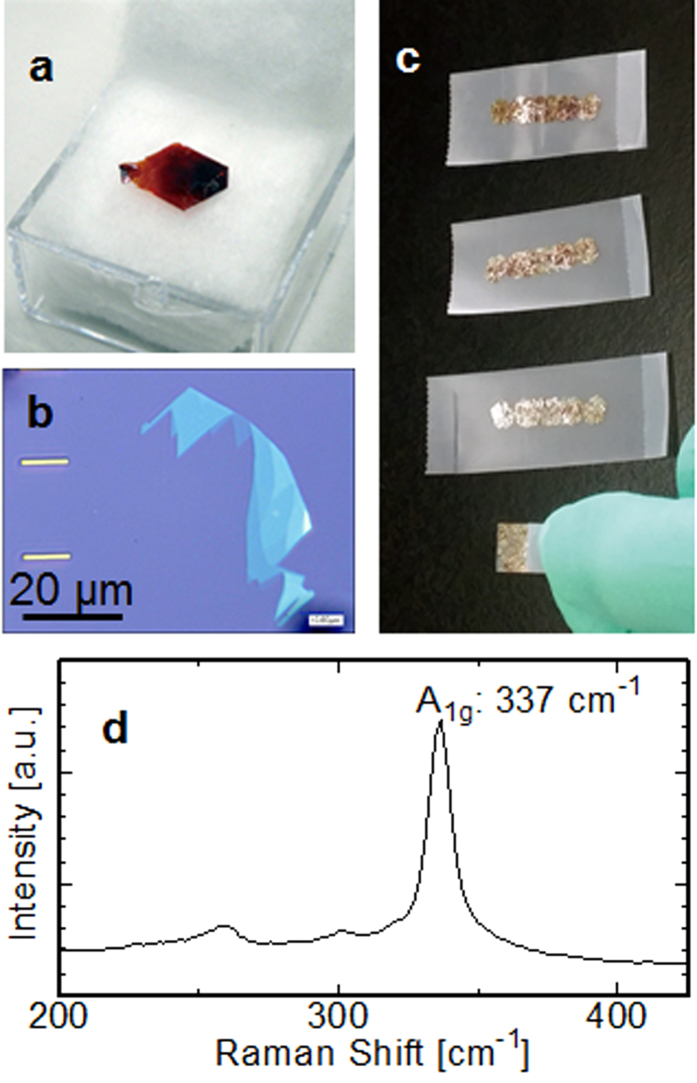
Micromechanical exfoliation of HfS_2_. (**a**) Single crystal piece of HfS_2_. (**b**) Mechanical cleavage of HfS_2_ using the ‘scotch tape’ method. The upper images show thicker layers, which were thinned by continuous exfoliations. (**c**) HfS_2_ transferred to 285-nm-thick SiO_2_ deposited on Si substrate. (**d**) Raman spectrum of the thin-film HfS_2_ on SiO_2_/Si substrate. Primary A_1g_ peak is observed at 337 cm^−1^ with some satellite peaks.

**Figure 4:**
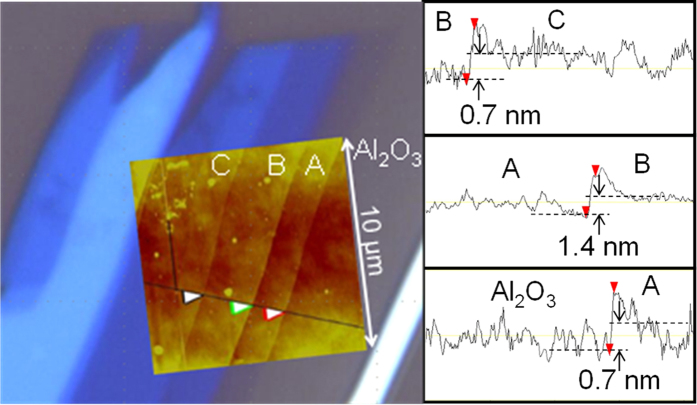
Optical image and AFM profiles of HfS_2_/Al_2_O_3_. Clear optical contrasts depending on the flake thickness are observed except in the thinnest region.

Figure 3(a) shows a piece of single-crystal HfS_2_, which is commercially available with high orientation and purity. We performed mechanical exfoliation using scotch tape to obtain thin-film HfS_2_ on the substrate. First, a small piece of HfS_2_ was spread on the tape and then cleaved several times to reduce its layer thickness to an average size, as shown in Fig. 3(b). Thickness-dependent colour contrast was distinctly observable. Figure 3(c) shows the optical image of the exfoliated thin-film HfS_2_ on a 285-nm-thick SiO_2_/Si substrate. Triangular or hexagonal shape often appeared in the flakes. These cleaved edges indicate the crystal orientation of the exfoliated HfS_2_. Incidentally, hexamethyldisilazane (HMDS) treatment helps in peeling a large size (>10 μm) atomically thin flake. The Raman spectrum of the thin HfS_2_ layers (<10 nm) on the SiO_2_/Si substrate with the excitation wavelength of 532 nm is shown in Fig. 3(d). The primary peak appeared at the Raman shift of approximately 337 cm^−1^, and it was consistent with the previous experimental report of bulk HfS_2_ that showed a first-order A_1g_ peak^28^. Satellite peaks at 260 and 321 cm^−1^ were also considered as E_g_ mode. These results indicate that single-crystal layers with well-aligned atoms remained intact during exfoliation and organic solvent cleaning. At this time, no remarkable change was observed from the bulk to the thin films. The visibility of the atomically thin HfS_2_ layers could be strongly dependent on the thickness and permittivity of the flake and the insulator, as reported in graphene^29^ and MoS_2_^30^. Figure 4 shows the optical microscope and the atomic force microscope (AFM) image (inset) with cut line profiles. The steps between the different contrast layers were approximately 0.7 nm/1.4 nm, and they appeared to be single/double atomic steps. On the 75-nm-thick Al_2_O_3_, the contrast in the single-layer HfS_2_ was too weak to form electrodes using the alignment technique. However, several layers of HfS_2_ were clearly observable by the optical microscope, and the thicknesses could be easily identified by the colour of the flakes.

**Figure 5:**
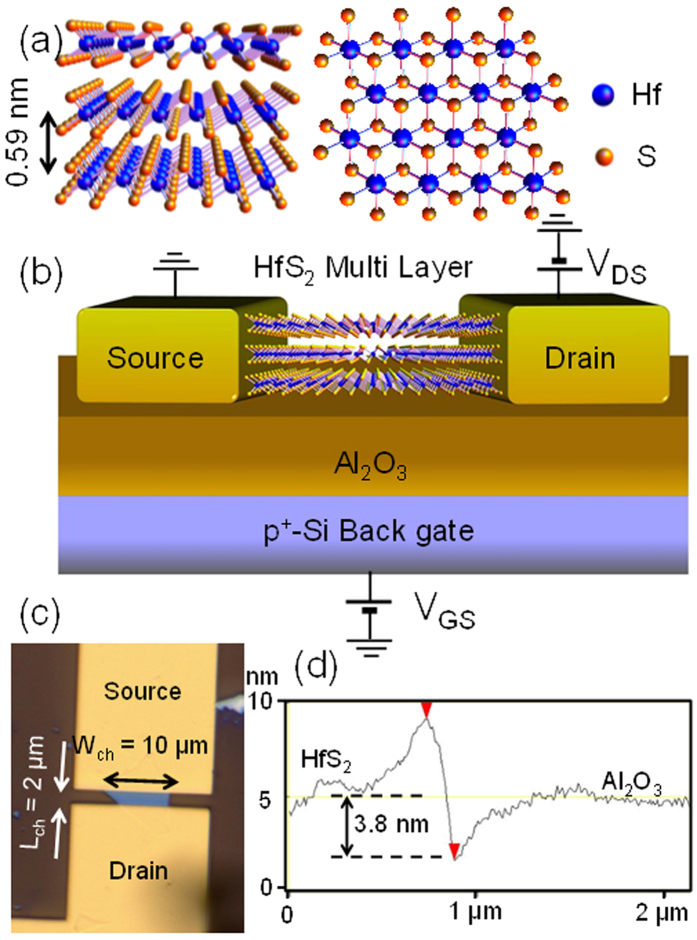
Device structure of fabricated HfS_2_ FET. (**a**) Crystal structure of HfS_2_. (**b**) Schematic of the device structure. The 3.8-nm-thick HfS_2_ layers were exfoliated on 75-nm-thick Al_2_O_3_, which was atomic layer-deposited on the degenerately doped p^+^-Si substrate. (**c**) Optical image of the fabricated HfS_2_ FET device. The channel length and width at the source edge are estimated to be approximately 2 and 10 μm, respectively. (**d**) Height profile of the channel layer obtained by AFM. The thickness is approximately 3.8 nm, which suggests that the channel contains approximately six atomic layers of HfS_2_.

HfS_2_ has a CdI_2_-like octahedral coordinated layered structure with an atomic layer thickness of 0.59 nm^31^ (Fig. 5(a)). The Hf atoms (blue spheres) are sandwiched by S atoms (yellow spheres), and the Hf atoms in each plane are stacked at the same position (1T, tetragonal symmetry). This crystal structure is different from that of MoS_2_, which typically has a trigonal prismatic coordinate with 2H symmetry. The schematic image of a fabricated FET with proper biases is shown in Fig. 5(b). The thin-body channel contains several atomic layers that were transferred onto the atomic layer deposited 75-nm-thick Al_2_O_3_/p^++^-Si(100) substrate. At first, we fabricated by used plasma-enhanced chemical vapour-deposited (PECVD) SiO_2_ (285 nm) as back-gate insulator for the first time. However, it could not work with clear current modulation by the back gate due to the small gate capacitance, high-density charge trapping and surface roughness caused due to PECVD SiO_2_ (please see supplementary Information Fig. S3). Au/Ti electrodes were fabricated on thin-film HfS_2_ as source and drain contacts. The source electrode was connected to the ground, and the drain electrode was positively biased with respect to the source (V_DS_). Back-gate voltage V_GS_ was applied to the p^++^-Si substrate. The optical microscope measurement suggested channel width and length of 10 μm (at the source edge) and 2 μm, respectively (Fig. 5(c)). From the cross-sectional height profile measured by AFM shown in Fig. 5(d), the thickness of the HfS_2_ channel was approximately 3.8 nm, which was composed of 6 ± 1 atomic layers.

### Back gate operation of the HfS_2_ FET

**Figure 6:**
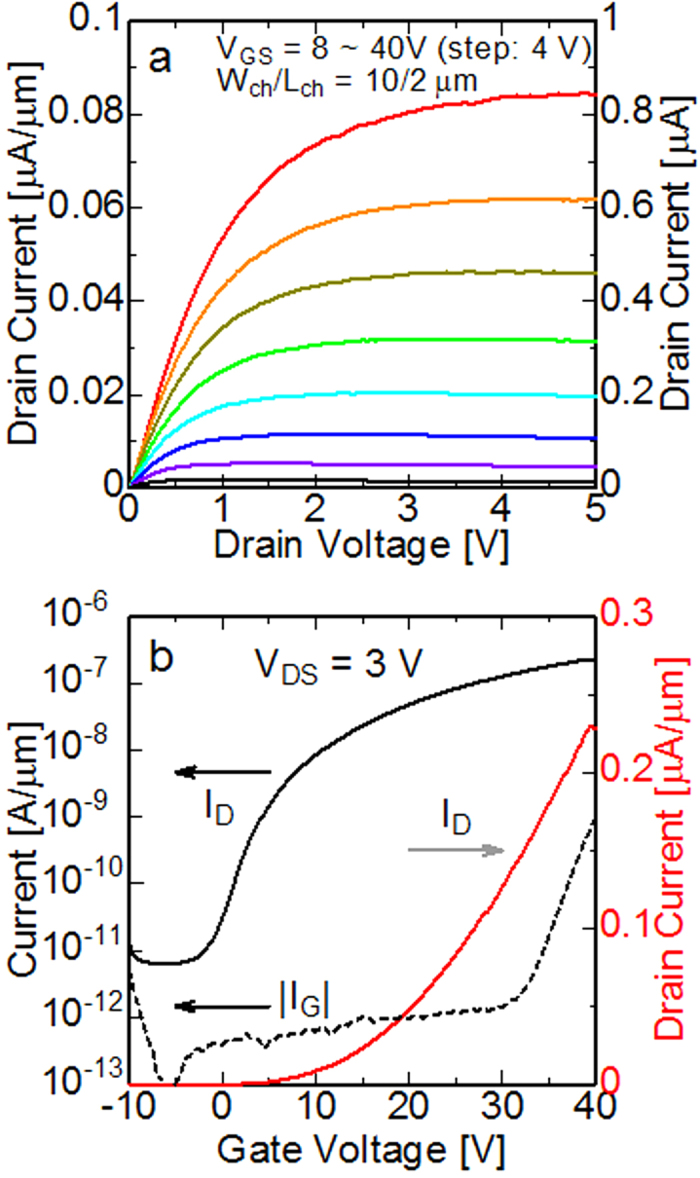
(**a**) Output characteristics with channel width and length of 10 and 2 μm, respectively, at RT. Current modulation property and robust saturation behaviour are observed. (**b**) Transfer characteristics with V_DS_ = 3 V. The maximum drain current obtained in this device is 0.2 μA/μm. The on/off current ratio in this voltage condition is over 10^4^. The gate leakage current is smaller than the drain current in the whole bias range but not negligible under a strong field. The FET operates as an enhancement-mode device.

Figure 6(a) shows the output characteristics of fabricated few-layer HfS_2_ FETs at RT. Clear saturation behaviour is observed in the I_D_–V_DS_ curves for all V_GS_ biases and V_DS_ sweep (0–5 V). The robust current saturation at high V_DS_ (5 V) indicates that the HfS_2_ bandgap is sufficient for short-channel FET fabrication. I_D_ continuously increments over the measured V_GS_ range. Unfortunately, our measurement system was not able to apply the voltage of over 40 V. A fully n-type enhancement mode operation with a threshold voltage greater than 8 V was observed. The I_D_–V_DS_ curves at the small bias regime showed linearity without offset bias. Although the instability of the I–V curves depends on the measurement cycles and sweep conditions, current modulations are constantly obtained for over a month in the atmosphere. The I_D_–V_GS_ (transfer) characteristics with logarithmic (left-hand side) and linear (right-hand side) scales for V_DS_ = 3 V are also shown in Fig. 6(b). A maximum drain current of 0.2 μA/μm was obtained at V_GS_ = 40 V. The on/off ratio was over 10,000 when the V_GS_ was varied from −5 V (off state) to 40 V (on state). These results were partially reported in ref. 32. The effective mobility of electrons was calculated to be around 0.1 cm^2^/V.s which is far smaller than the theoretical expectation. It was probably limited by many intrinsic and parasitic components such as the carrier scattering, quality of flakes, contact resistance and charge trapping. This device had a large hysteresis (~15 V) (please see supplementary Information Fig. S4), which could have been caused by the response of the traps at the interface between HfS_2_ and Al_2_O_3_ or border/oxide traps in the bulk Al_2_O_3_. The gate leakage current I_G_ is also plotted in logarithmic scale, which shows that I_G_ is comparable with I_D_ for higher values of V_GS_. Therefore, the on current and other performance of the present device can be improved by efficient modulation of the surface potential using a thinner gate dielectric with good interface properties.

### Electric double layer transistor with HfS_2_ channel

**Figure 7:**
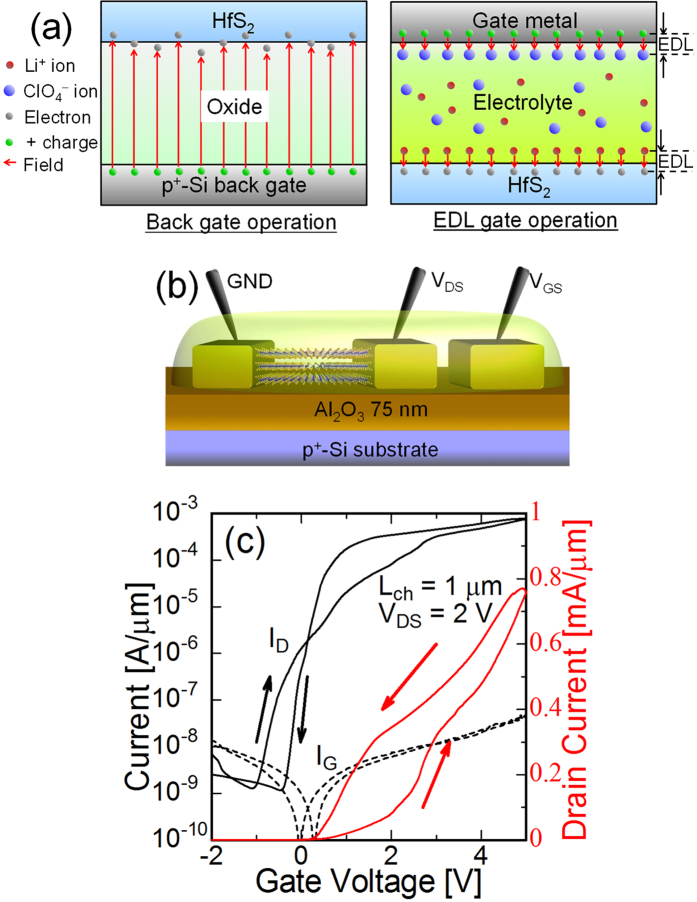
Schematics and I-V characteristics of an electric double layer transistor. (**a**) Carrier density modulation by the back gate and EDL. (**b**) Fabricated EDL transistor. PEO:LiClO_4_ electrolyte gel is used for the EDL gate. (**c**) Transfer characteristics at V_DS_ = 2 V. The maximum drain current is 0.75 mA/μm at V_GS_ = 5 V.

The gate-induced carrier density in the HfS_2_ channel was limited by the back-gate properties such as interface/bulk charge trapping. To evaluate the potential of HfS_2_, we introduced the electric double-layer (EDL) gate structure^33^ for device measurement. Schematics of the carrier density modulation by back gate and EDL are shown in Fig. 7(a). In the case of Al_2_O_3_ back gate, the gate electric fields pass through the thick insulator and are partially terminated at the immobile charges. On the other hand, in the case of EDL operation, anions and cations (ClO_4_^−^ and Li^+^ respectively) can freely move in the electrolyte. These ions form the EDL with carriers in semiconductors and metals, and the electric field is localized near the surface (~5 nm). Therefore, the large gate capacitance was expected without the charge trapping. Moreover, the surface of the TMD does not have both dangling bond and disorder of atoms. The combination of TMD and electrolyte gate can potentially realise an ideal interface between the semiconductor and dielectric and efficient carrier modulation^34^. Figures 7(b,c) show the schematic device structure and transfer characteristics, respectively, of a fabricated HfS_2_ EDL transistor. LiClO_4_/PEO mixture was employed as an electrolyte gel for gate modulation. This time, the gate-contact electrode was far from the channel region and its area was not sufficiently large. Therefore, the effective gate voltage was smaller than the measured value (probably half or less). The measured drain current was over 0.75 mA/μm at V_DS_ = 2 V and V_GS_ = 5 V at RT. The I_DS_ significantly increased from the back-gate operation and was notably high at the TMD channel in spite of the non-scaled channel length (1 μm). The maximum current density of a conventional TMD FET with a solid gate structure is approximately 0.5 mA/μm for MoS_2_, and it is limited by the contact resistance between MoS_2_ and the metal. The high drain current observed in this study indicates the superior potential of the low contact resistivity of HfS_2_. The relative hysteresis against the bias range was significantly reduced by the EDL gate. It means that the density of trap charges in EDL gate was far smaller than that of Al_2_O_3_ back gate. Improvement in back gate insulator would increase the drain current and reduce the hysteresis. The remaining hysteresis of the EDL transistor appeared to be caused by the response time of the anions and cations in the electrolyte. According to the magnitude of the gate current, the drain current might not depend on the electrochemical reaction at the electrode surface. The effective mobility of EDL device was roughly estimated to be 45 cm^2^/Vs from the peak g_m_ by the assumption that the permittivity of the electrolyte is 5ε_0_ and the EDL thickness is 1 nm. It was clearly improved from the back-gate operation. However, the mobility is still smaller than the expected value (>1,000 cm^2^/V·s). One of the reasons can be attributed to the gate structure not being large enough for the voltage drop in the EDL to be negligible at the gate side. Therefore, the actual gate voltage for the channel EDL is less than the applied value. The accurate estimation of the contact resistance of Ti/HfS_2_ contact was difficult for this device. According to the maximum value of I_D_ and applied V_DS_, on resistance was 2.7 kΩ·μm at most. The on resistance is comparable to the typical contact resistance of other many materials. Further evaluations and improvements is required to verify the potential of the HfS_2_ as the channel material.

## Conclusion

In conclusion, we have demonstrated the fabrication and I–V characteristics of few layer HfS_2_ FETs. Mechanical exfoliation using scotch tape provided an atomically thin HfS_2_ single-crystal layer with reasonable Raman spectrum and optical contrast with several number of layers. For a channel thickness of 3.8 nm and back gate bias, robust saturation behaviour and a drain current of 0.2 μA/μm were observed with high on/off current ratio (>10^4^). Moreover, EDL gate operation obtained the significant increase of the drain current (~0.75 mA/μm). This improved property seemed to indicate the intrinsic performance of HfS_2_. These results provided basic information of HfS_2_ as electron devices, and the attractive properties considered as significant for ultra-low power applications were experimentally demonstrated.

## Materials and Methods

### Fabrication Process

Initially, 75-nm-thick Al_2_O_3_ was deposited on p^++^-Si (ρ < 0.001 Ω∙cm) by thermal atomic layer deposition system (Ultratech/Cambridge Nanotech Savannah S100) as a back-gate insulator. Next, HfS_2_ flakes were mechanically exfoliated from the highly oriented crystal with high purity (99.995%, HQ Graphene) using the scotch tape method and transferred on the Al_2_O_3_ surface. The Al_2_O_3_ surface was passivated by HMDS to prepare a hydrophobic surface suitable for bonding with HfS_2_ (please see supplementary Information Fig. S2). In this study, the exfoliated HfS_2_ flakes for both the channel and contact regions were not intentionally doped. After the optical identification of the transferred flakes, the alignment exposure of the source and drain electrodes was carried out by electron beam (EB) lithography (Crestec CABL-9000) using PMMA (polymethyl methacrylate). Then, Ti(20 nm)/Au(100 nm) were EB-evaporated and lifted off. Finally, back-gate contact was formed by EB evaporation of Cr (20 nm) and Au (100 nm).

### Measurement Systems

The thickness of the HfS_2_ thin films were evaluated by AFM (Veeco Nanoscope III). All DC characteristics reported in this letter were measured by an Agilent 4155B semiconductor parameter analyser.

## Additional Information

**How to cite this article**: Kanazawa, T. *et al.* Few-layer HfS_2_ transistors. *Sci. Rep.* **6**, 22277; doi: 10.1038/srep22277 (2016).

## Supplementary Material

Supplementary Information

## Figures and Tables

**Figure 1:**
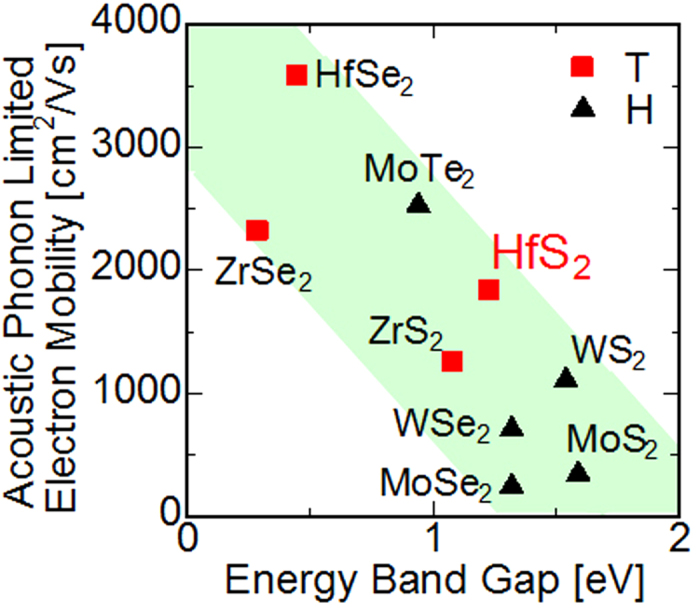
Mobility and Bandgap plot of typical TMDs. The acoustic phonon-limited electron mobilities and energy band gap calculated in refs. 10 and 11 are plotted. The patterns indicate the coordinate structure (T: octahedral coordination and H: triangle prism coordination).
