# Supplementary material for: Few-layer HfS2 transistors
Source: Sci Rep. 2016 Mar 1;6:22277. doi: 10.1038/srep22277 (PMC4772098; doi:10.1038/srep22277)
Supplement: Supplementary Information [file srep22277-s1.pdf]

Supplementary Information for ‘Fabrication of a few-layer HfS_2 transistor.’

Toru Kanazawa[1*], Tomohiro Amemiya[2,3*], Atsushi Ishikawa[3,4], Vikrant Upadhyaya[1], Kenji Tsuruta[4], Takuo Tanaka[3], and Yasuyuki Miyamoto[1]

[1]Department of Physical Electronics, Tokyo Institute of Technology, 152-8552 Japan
[2]Quantum Nano Electronics Research Center, Tokyo Institute of Technology, 152-8552 Japan
[3]Metamaterial Laboratory, RIKEN, 351-0198, Japan
[4]Department of Electrical & Electronic Engineering, Okayama University, 700-8530, Japan

*e-mail: kanazawa.t.aa@m.titech.ac.jp, amemiya.t.ab@m.titech.ac.jp

Theoretical calculation of the ballistic current in single-layer HfS_2

We used the simple ballistic model to evaluate the transport properties of single-layer HfS_2. 2D k-plane with 3-fold valleys at M points were considered. Each valley assumed to have the parabolic and ellipsoidal conduction band with the E-k relation of

$$E = \frac{\hbar^2}{2}\left(\frac{{k_l}^2}{m_l} + \frac{{k_t}^2}{m_t}\right).$$

where k_l, m_l, k_t, m_t are the wavenumbers and effective masses for longitudinal (transverse) direction.

The drain bias is high enough to ignore the drain injection. Fermi energy was only controlled by the gate bias not affected by the drain bias.

The small current towards the drain, is given by

$$dJ = q\; dn\; v_{channel},$$

where electron density dn and velocity along the channel $v_{channel}$ are calculated as follows

$$dn = \frac{2}{(2\,\pi)^2}\,\frac{1}{1+\exp(\frac{E_F-E}{k_B T})},$$

$$v_{channel} = \frac{\hbar\,(k_l \cos\alpha + k_t \sin\alpha)}{m_c}.$$

m_c is the direction dependent effective mass and α is the angle between the channel direction and M-Γ direction. Figure 2 (a) indicates the dJ distribution in 2D k-plane by the contour plot.

Next, small current dJ was integrated for k-space and summed for 3 valleys. For the ballistic limit, all electrons at the potential bottleneck have positive wave number. Then, electron states with negative velocity against the channel direction were assumed to be empty.

Finally, gate voltage was defined as the sum of the Fermi energy shift and the voltage applied to the gate insulator. Applied Voltage to the gate insulator was defined as $V_{ox} = \frac{q\,N_{sheet}}{\varepsilon_{ox}\,\varepsilon_0} t_{ox}$. Then, total gate voltage is $V_G = \frac{E_F - E_0}{q} + \frac{q\,N_{sheet}}{\varepsilon_{ox}\,\varepsilon_0} t_{ox}$. This E_0 was determined as the Fermi Energy for the calculated current density is set at 100 nA/μm (which is the off current for high performance logic circuits required by the International Technology Roadmap for Semiconductors).

Thickness and contrast evaluation of HfS_2 layers

We confirmed the layered exfoliation of HfS_2 on SiO_2 substrate. The optical image had a clear discrete colour variation from blue (thin) to white (thick). The CCD count rate along the white dotted line from A to B was also described. The counting values decreased in incremental steps from the substrate to thicker HfS_2.

The AFM measurement showed triangular structure, and the smallest step size was estimated to be less than 0.9 nm. We could reasonably consider that the step indicate the single layer of HfS_2.

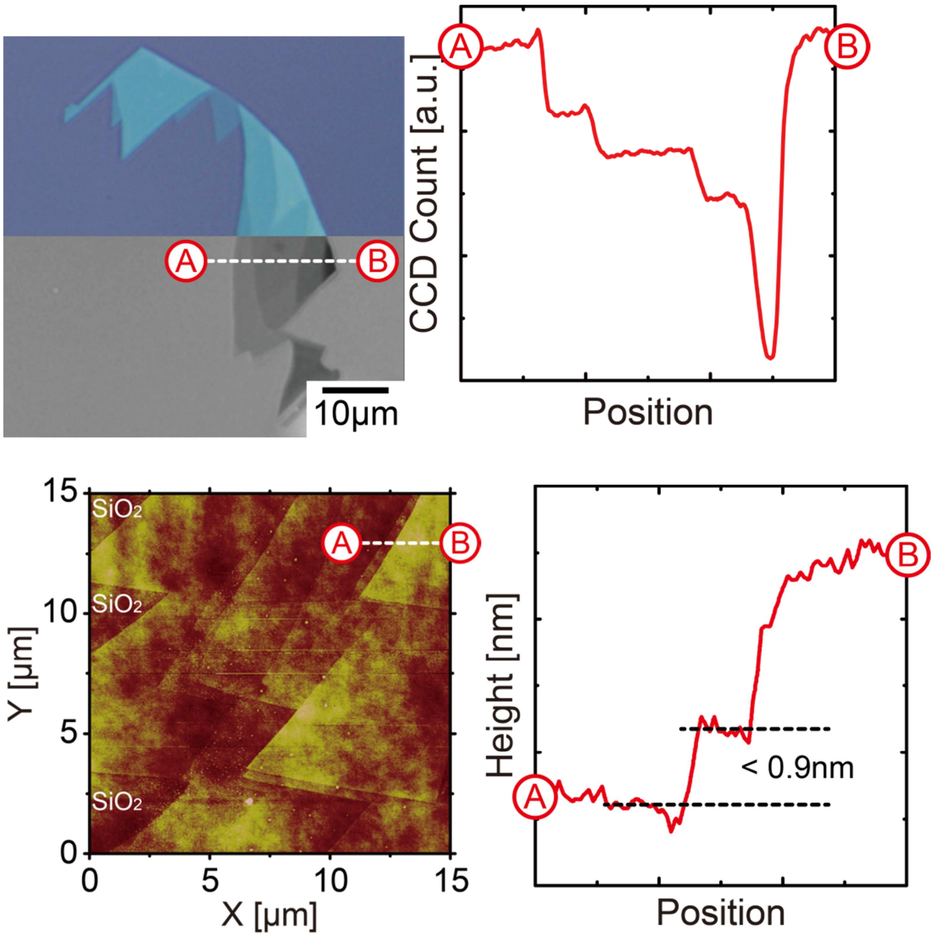

Figure S1. Optical contrast and monolayer thickness of HfS_2/SiO_2 285 nm/Si. a, Optical image and contrast profile. b, AFM image and cut line profile.

Hydrophyte evaluation of HfS_2

The contact angles of water drops for HfS_2, highly oriented pyrolytic graphene, SiO_2, O_3-cleaned SiO_2, and HMDS-self-assembled monolayer (SAM)-treated SiO_2 were evaluated. The HfS_2 crystal surface showed strong hydrophobicity similar to graphene, which means that HMDS-SAM treatment is suitable for transferring not only graphene but also HfS_2.

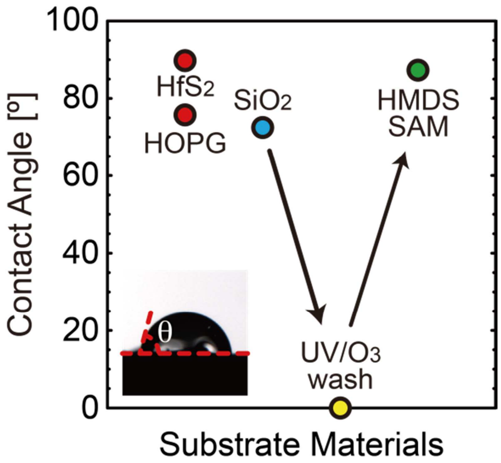

Figure S2. Contact angle evaluation of HfS_2, graphene, SiO_2, and HMDS-SAM-treated SiO_2

Current properties of HfS_2 FET with SiO_2/Si back gate

The current properties of HfS_2 FET with SiO_2/Si back gate operation are shown below. The on/off ratio is only 10 for a bias range of 0–20 V and the maximum drain current is 10 pA/μm at V_{GS} = 20 V. This inferior modulation property of V_{GS} is probably due to the defect traps and rough surface of the SiO_2 back-gate insulator deposited by plasma-enhanced chemical vapour deposition. Following these results, the device was fabricated with ALD Al_2O_3 to improve the quality of the back gate insulator and that device has been reported mainly in this report.

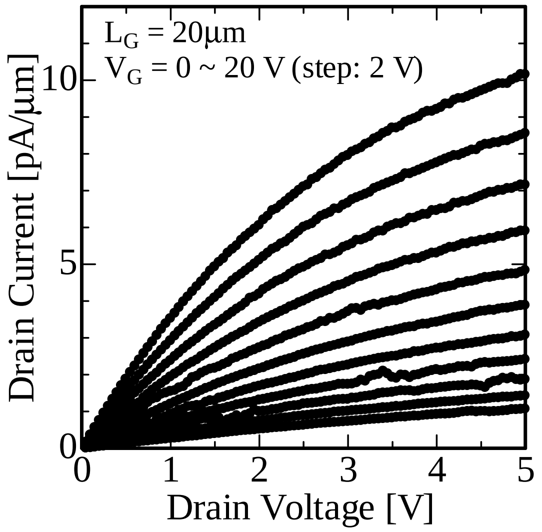

Figure S3. I_D–V_{DS} characteristics of the HfS_2 FET formed with (SiO_2)/Si back gate.

Hysteresis characteristics of HfS_2 MOSFET

Here, we discuss the hysteresis characteristics of the HfS_2 FET for back-gated operation. Figure S4 shows the logarithmic plot of the I_D–V_{GS} characteristics. The ΔV_{GS} shift in the subthreshold region in the hysteresis characteristics was approximately 15 V.

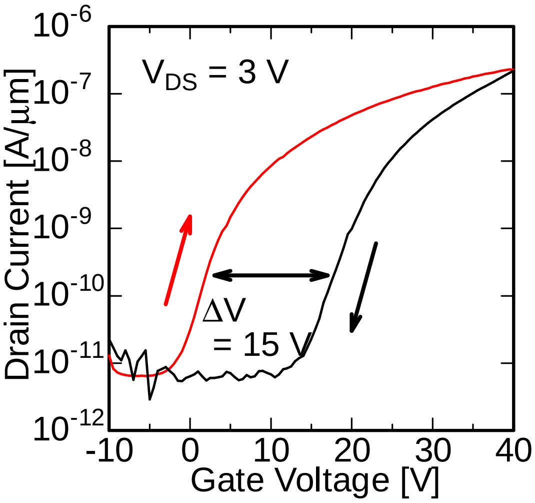

Figure S4. Double-sweep transfer characteristics of HfS_2 transistor.
